# Cromolyn Reduces Levels of the Alzheimer’s Disease-Associated Amyloid β-Protein by Promoting Microglial Phagocytosis

**DOI:** 10.1038/s41598-018-19641-2

**Published:** 2018-01-18

**Authors:** Can Zhang, Ana Griciuc, Eloise Hudry, Yu Wan, Luisa Quinti, Joseph Ward, Angela M. Forte, Xunuo Shen, ChongZhao Ran, David R. Elmaleh, Rudolph E. Tanzi

**Affiliations:** 1 Genetics and Aging Research Unit, MassGeneral Institute for Neurodegenerative Diseases (MIND), Department of Neurology, Massachusetts General Hospital and Harvard Medical School, Charlestown, MA 02129–2060 USA; 2 Alzheimer’s Disease Research Unit, MassGeneral Institute for Neurodegenerative Diseases (MIND), Department of Neurology, Massachusetts General Hospital and Harvard Medical School, Charlestown, MA 02129-2060 USA; 3 Department of Radiology, Massachusetts General Hospital and Harvard Medical School, Charlestown, MA 02129-2060 USA

## Abstract

Amyloid-beta protein (Aβ) deposition is a pathological hallmark of Alzheimer’s disease (AD). Aβ deposition triggers both pro-neuroinflammatory microglial activation and neurofibrillary tangle formation. Cromolyn sodium is an asthma therapeutic agent previously shown to reduce Aβ levels in transgenic AD mouse brains after one-week of treatment. Here, we further explored these effects as well as the mechanism of action of cromolyn, alone, and in combination with ibuprofen in APP^Swedish^-expressing Tg2576 mice. Mice were treated for 3 months starting at 5 months of age, when the earliest stages of β-amyloid deposition begin. Cromolyn, alone, or in combination with ibuprofen, almost completely abolished longer insoluble Aβ species, i.e. Aβ40 and Aβ42, but increased insoluble Aβ38 levels. In addition to its anti-aggregation effects on Aβ, cromolyn, alone, or plus ibuprofen, but not ibuprofen alone, increased microglial recruitment to, and phagocytosis of β-amyloid deposits in AD mice. Cromolyn also promoted Aβ42 uptake in microglial cell-based assays. Collectively, our data reveal robust effects of cromolyn, alone, or in combination with ibuprofen, in reducing aggregation-prone Aβ levels and inducing a neuroprotective microglial activation state favoring Aβ phagocytosis versus a pro-neuroinflammatory state. These findings support the use of cromolyn, alone, or with ibuprofen, as a potential AD therapeutic.

## Introduction

Alzheimer’s disease (AD) is an insidious neurodegenerative disorder and the major cause of dementia in the elderly, with no effective disease-modifying therapy currently available^1^. Genetic, biochemical, molecular biological, and pathological evidence supports the amyloid hypothesis, positing that the excessive accumulation of Aβ is the primary pathological event leading to neurofibrillary tangles (NFT), neurodegeneration, and neuroinflammation in AD^2–4^. Aβ42 and Aβ40 are the two primary Aβ species, with Aβ42 the more prevalent isoform in β-amyloid plaques^3–5^. Aβ is deposited early in the disease process decades prior to symptoms, suggesting the need for secondary prevention of AD in therapies aimed at β-amyloid^2^. In contrast, neuroinflammation occurs throughout the course of the disease, particularly in later stages. Decreasing Aβ42 and NFT levels, and reducing neuroinflammation are all considered potentially effective means for preventing and treating AD.

Cromolyn is a small molecule approved for the treatment of asthma. Cromolyn has structural similarity to fisetin, an anti-Aβ aggregation molecule, and has been previously shown to significantly inhibit Aβ aggregation *in vitro*^6^. Additionally, in the transgenic APPswe/PS1ΔE9 mouse model, expressing the familial amyloid precursor protein (APP) Swedish mutation and the presenilin (PS1) mutation that lacks the exon 9, intraperitoneal injection of cromolyn administrated for a week, previously led to a significant and robust (>50%) reduction in soluble and insoluble Aβ levels^6^. Aβ-mediated neurodegeneration and NFT formation both contribute to microglial cell-mediated neuroinflammatory responses in the AD brain^7,8^. Depending on activation state, microglia can either phagocytose Aβ and display an anti-inflammatory phenotype or secrete pro-inflammatory cytokines and free radicals causing neuroinflammation^9,10^. Previous studies suggest that microglia transition from a largely anti-inflammatory/pro-phagocytic to a pro-inflammatory/neurotoxic activation state during AD progression^11^. Pro-inflammatory cytokines produced in response to Aβ accumulation downregulate genes involved in Aβ clearance and promote Aβ plaque deposition, thereby contributing to neurodegeneration. Neuroinflammation can also promote vascular leakage through the blood-brain barrier^12^ and promote the additional generation of Aβ and NFT^13,14^. Thus, neuroinflammation, in combination with Aβ-mediated tauopathy and neurodegeneration, leads to a vicious cycle resulting in progressive neuronal cell death, synapse loss, and, ultimately, dementia. Therefore, compounds that induce microglial cells into a pro-phagocytic activation state could make useful AD therapeutics.

Here, we investigated whether chronic treatment with cromolyn, alone, or in combination with ibuprofen (a non-steroidal anti-inflammatory drug and cox1/cox2 non-specific modulator) affects the levels of Aβ in a well-characterized transgenic AD mouse model, the APPswe Tg2576^15,16^. Five month old Tg2576 mice were treated with cromolyn, alone, or in combination with ibuprofen via intraperitoneal injection for 3 months, an age at which major plaque accumulation is not yet observed. We found that cromolyn, alone, and in combination with ibuprofen, almost completely abolished the levels of brain TBS-insoluble (formic acid extracted) Aβ, which drives deposition of β-amyloid. We also showed that cromolyn (alone and in combination with ibuprofen) increased the number of Iba-1 positive microglia surrounding β-amyloid plaques as compared to vehicle-treated animals. Finally, we also employed a microglial cell culture model to show that cromolyn (alone and with ibuprofen) promoted a microglial activation state defined by enhanced phagocytosis of Aβ42. Collectively, these findings strongly support the potential use of cromolyn and cromolyn plus ibuprofen as therapeutics for AD-related β-amyloid deposition and in modulating microglial activation state in a direction toward phagocytosis and, therefore, away from neuroinflammation.

## Results

We carried out animal studies using the well-characterized APP^Swedish^-expressing Tg2576 mice. 3-month old Tg2576 mice were acclimatized for 2 months and then randomly assigned to a total of 7 treatment groups, including 6 drug-treatment groups and one vehicle-treatment group (Table 1). Each group received one dose of cromolyn (1.0 or 3.15 mg/kg), ibuprofen (0.5 or 2.0 mg/kg), cromolyn plus ibuprofen (n = 8–10/group), or PBS (vehicle; n = 10). The drugs were prepared in sterile PBS and administered by IP, based on 0.1 mL/30 g body weight. The treatments were performed 3 times per week for 12 weeks in total. The body weight was measured 2 weeks after the initial treatment and once per week afterwards. We found that all treatments were well tolerated, and there were no significant differences in body weight among the groups throughout the treatment period (Supplementary Figure 1). All treated mice were sacrificed at 8-month old and tissues were harvested and processed for post-mortem analysis.

**Table 1:** Experimental groups and treatments.

Group (n)	Treatments	Dose (mg/kg)	Schedule	Duration
1 (10)	Vehicle	PBS	3 X /week; ip	12 weeks
2 (9)	Cromolyn (low-dose)	1	3 X /week; ip	12 weeks
3 (8)	Cromolyn (high-dose)	3.15	3 X /week; ip	12 weeks
4 (10)	Ibuprofen (low-dose)	0.5	3 X /week; ip	12 weeks
5 (10)	Ibuprofen (high-dose)	2	3 X /week; ip	12 weeks
6 (10)	Combined low-dose (Cromolyn + Ibuprofen)	1 + 0.5 (respectively)	3 X /week; ip	12 weeks
7 (9)	Combined high-dose (Cromolyn + Ibuprofen)	3.15 + 2 (respectively)	3 X /week; ip	12 weeks

Three-month old Tg2576 mice were randomly assigned to 7 treatment groups and then acclimatized for 2 months at the study facility, followed by vehicle or drug treatment. The first was control group receiving vehicle (PBS) treatment (n = 10). The drug-treatment groups were divided to low and high-dose groups, receiving either ibuprofen, cromolyn or their combination (n = 8–10). All 7-group mice were IP injected based on 0.1 mL/30 g body weight for 3 times per week for 12 weeks and were sacrificed at 8-month old. Tissues were harvested and processed for post-mortem analysis.

### Effects of Cromolyn and Ibuprofen on Aβ levels in Tg2576 Mice

**Figure 1:**
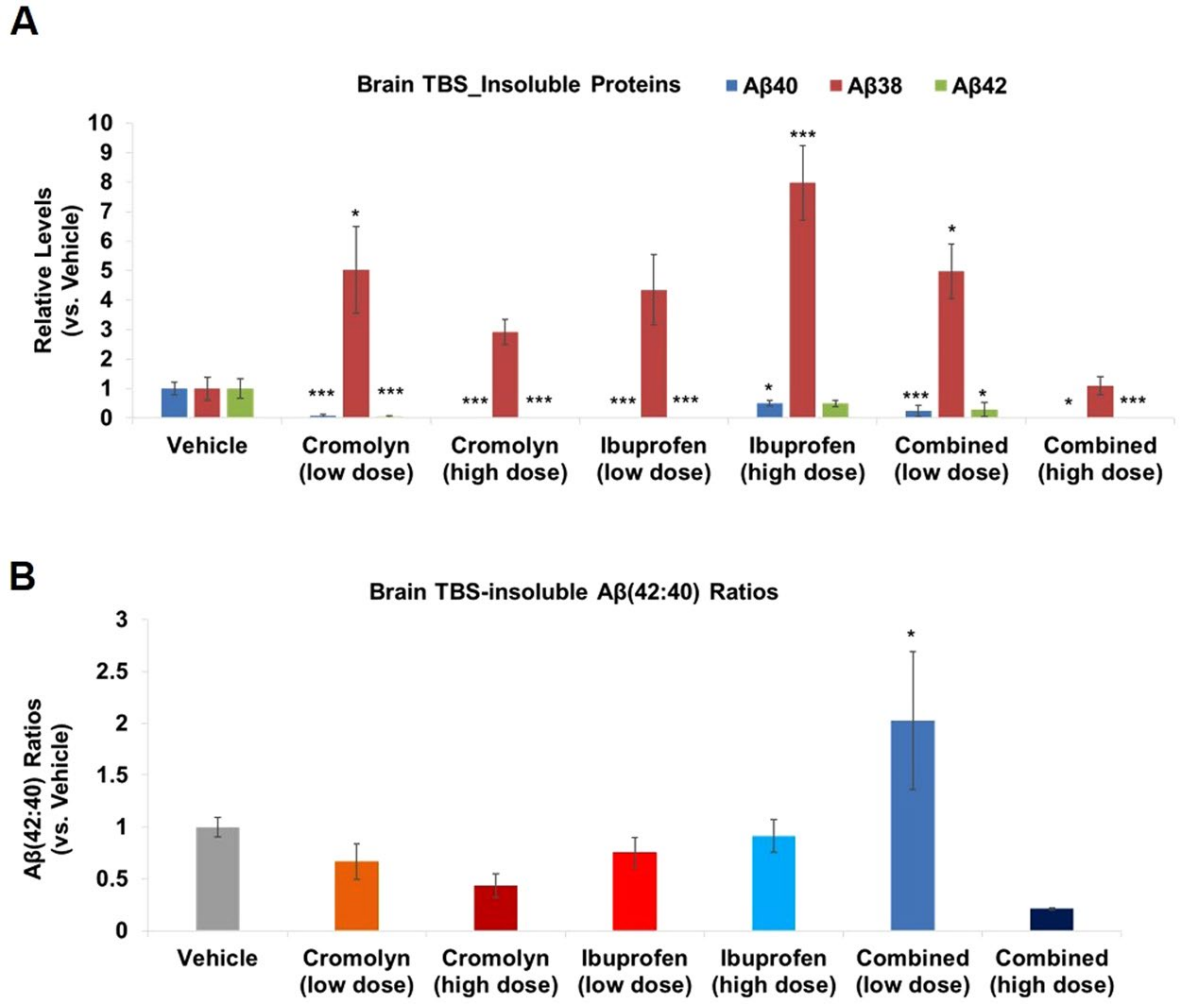
Cromolyn and/or ibuprofen robustly affected Aβ levels and Aβ42:Aβ40 ratios in brain TBS-insoluble samples. (**A**,**B**) MesoScale Aβ-triplex analyses were applied to brain TBS-insoluble samples. Differences in Aβ levels (**A**) and Aβ(42:40) ratios (**B**) were assessed comparing various treatment groups to vehicle. Mean ± SEM; n ≥ 8 (Table 1); *p < 0.05; ***p< 0.001 (vs. vehicle); one-way ANOVA with Dunnett’s multiple comparison test. Abbreviations: Aβ(42:40): Aβ42: Aβ40.

We first investigated whether cromolyn, ibuprofen, or cromolyn plus ibuprofen influenced brain Aβ levels in the TBS-insoluble (formic acid extracted) protein fraction. In the TBS-insoluble fraction, both low and high-dose cromolyn almost completely abolished both Aβ40 and Aβ42 species (p < 0.001; vs. vehicle; Fig. 1A**;** Supplementary Figure 2A), while robustly increasing Aβ38 levels. Specifically, low-dose cromolyn decreased Aβ40 and Aβ42 levels by 92.4% (p < 0.001) and 94.8% (p < 0.001), respectively, and increased Aβ38 levels by 402.0% (p < 0.05) compared to vehicle. High-dose cromolyn decreased Aβ40 and Aβ42 levels by 98.7% (p < 0.001) and 99.6% (p < 0.001), respectively, and increased Aβ38 levels by 191.6% as compared to vehicle. We also found that low and high-dose ibuprofen alone decreased both Aβ40 and Aβ42 levels (p < 0.001 and p < 0.05, respectively; Fig. 1A**;** Supplementary Figure 2A), while robustly increasing Aβ38 levels (p < 0.001 for the high-dose). Combined low-dose cromolyn and ibuprofen also led to a significant decrease in Aβ40 and Aβ42 levels (p < 0.001 and p < 0.05, respectively; vs. vehicle; Fig. 1A**;** Supplementary Figure 2A) and a marked increase in Aβ38 levels (p < 0.05; vs. vehicle; Fig. 1A**;** Supplementary Figure 2A**)**. Finally, combined high-dose cromolyn and ibuprofen displayed a significant decrease in both Aβ40 and Aβ42 levels (p < 0.05 and p < 0.001, respectively; vs. vehicle; Fig. 1A), but with no significant change in Aβ38 levels as compared to vehicle (Fig. 1A**;** Supplementary Figure 2A**)**.

We next analyzed the effects of cromolyn and ibuprofen, alone, or in combination, on the Aβ42:Aβ40 ratio in brain TBS-insoluble fractions as compared to vehicle. Neither cromolyn nor ibuprofen, alone, affected Aβ42:Aβ40 ratios vs. vehicle (Fig. 1B**;** Supplementary Figure 2B). Interestingly, combined low-dose cromolyn with ibuprofen led to an increased Aβ42:Aβ40 ratio (p < 0.05; vs. vehicle; Fig. 1B**;** Supplementary Figure 2B**)**. However, this did not appear to be dose-dependent since combined high-dose cromolyn with ibuprofen did not significantly affect the Aβ42:Aβ40 ratio vs. vehicle (Fig. 1B; Supplementary Figure 2B).

**Figure 2:**
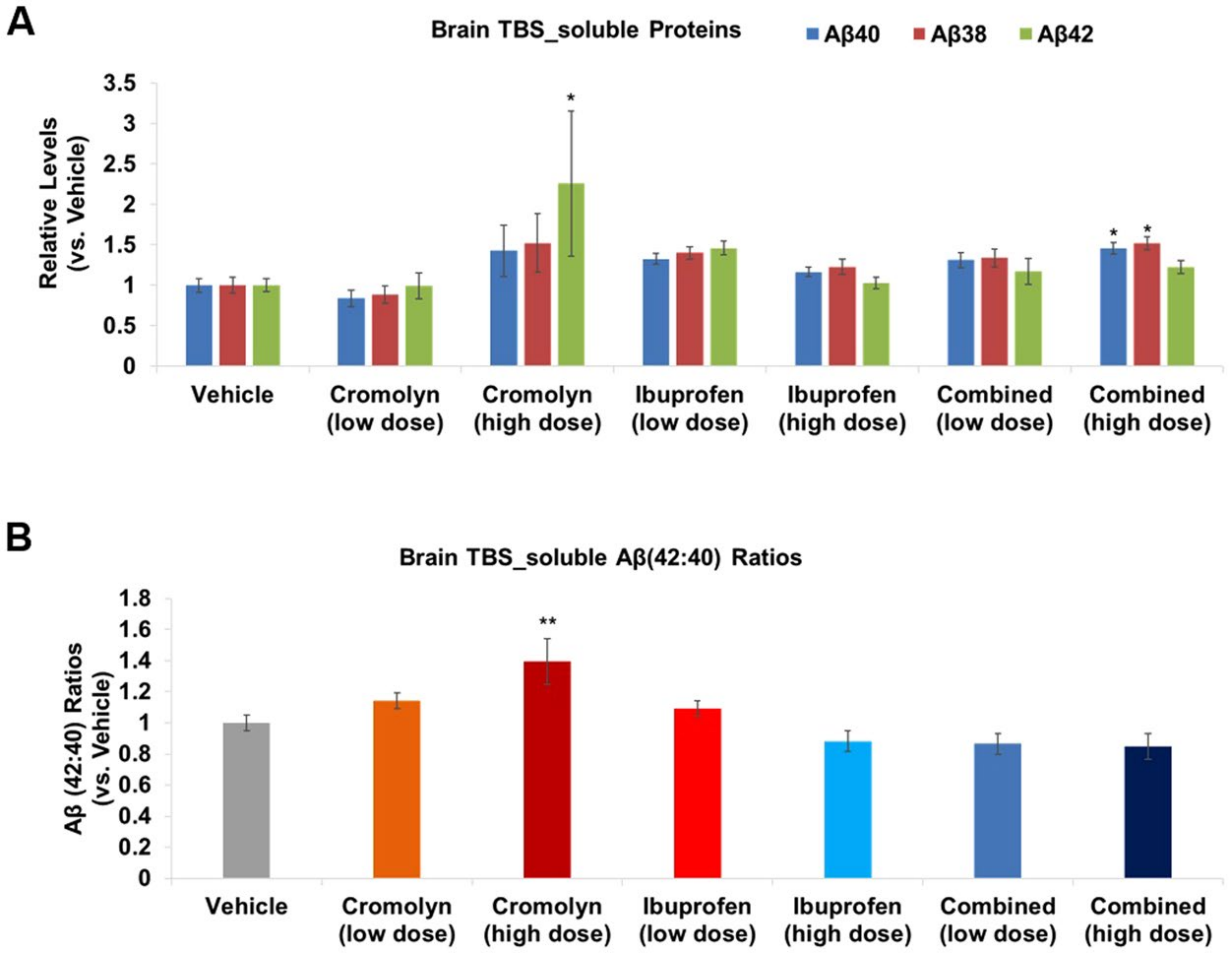
High dose cromolyn and combined high dose cromolyn with ibuprofen upregulated Aβ levels in brain TBS-soluble samples; High dose cromolyn elevated Aβ42:Aβ40 ratios in brain TBS-soluble samples. (**A**,**B**) MesoScale Aβ-triplex analyses were applied to brain TBS-soluble samples. Differences in Aβ levels (**A**) and Aβ42:Aβ40 ratios (**B**) were analyzed comparing various treatment groups to vehicle. Mean ± SEM (normalized to vehicle); n ≥ 8 (Table 1); *p < 0.05; **p < 0.01 (vs. vehicle); one-way ANOVA with Dunnett’s multiple comparison test.

We next investigated the effects of cromolyn and ibuprofen, alone, or in combination on brain TBS-soluble protein fractions. Neither cromolyn, nor ibuprofen alone, signicantly affected Aβ levels, although high-dose cromolyn increased levels of soluble Aβ42 (by 126.0%; p < 0.05; Fig. 2A; Supplemental Figure 2C), as well as the soluble Aβ42:Aβ40 ratios (Fig. 2B; Supplemental Figure 2D**)**. In addition, combined high-dose cromolyn and ibuprofen significantly increased levels of soluble Aβ40 and Aβ38 levels (p < 0.05; Fig. 2A**;** Supplemental Figure 2C). With regard to effects of cromolyn and ibuprofen, alone, or in combination on Aβ42:Aβ40 ratios in TBS-soluble fractions, high-dose cromolyn, alone, significantly increased Aβ42:Aβ40 ratios as compared to vehicle (by 40.0%; p < 0.01; Fig. 2B; Supplemental Figure 2D).

**Figure 3:**
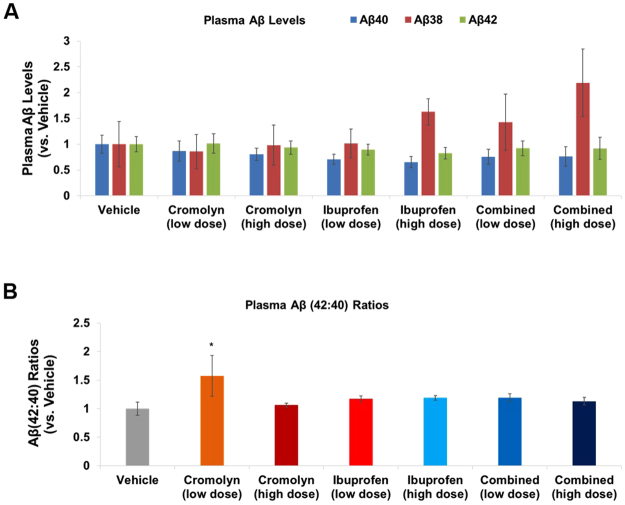
Low dose cromolyn increased plasma Aβ42:Aβ40 ratios. (**A**,**B**). MesoScale Aβ-triplex analyses were applied to plasma samples. Differences in Aβ levels (**A**) and Aβ42:Aβ40 ratios (**B**) in plasma were analyzed comparing various treatment groups to vehicle. Mean ± SEM; n ≥ 8 (Table 1); *p < 0.05 (vs. vehicle), one-way ANOVA with Dunnett’s multiple comparison test.

**Figure 4:**
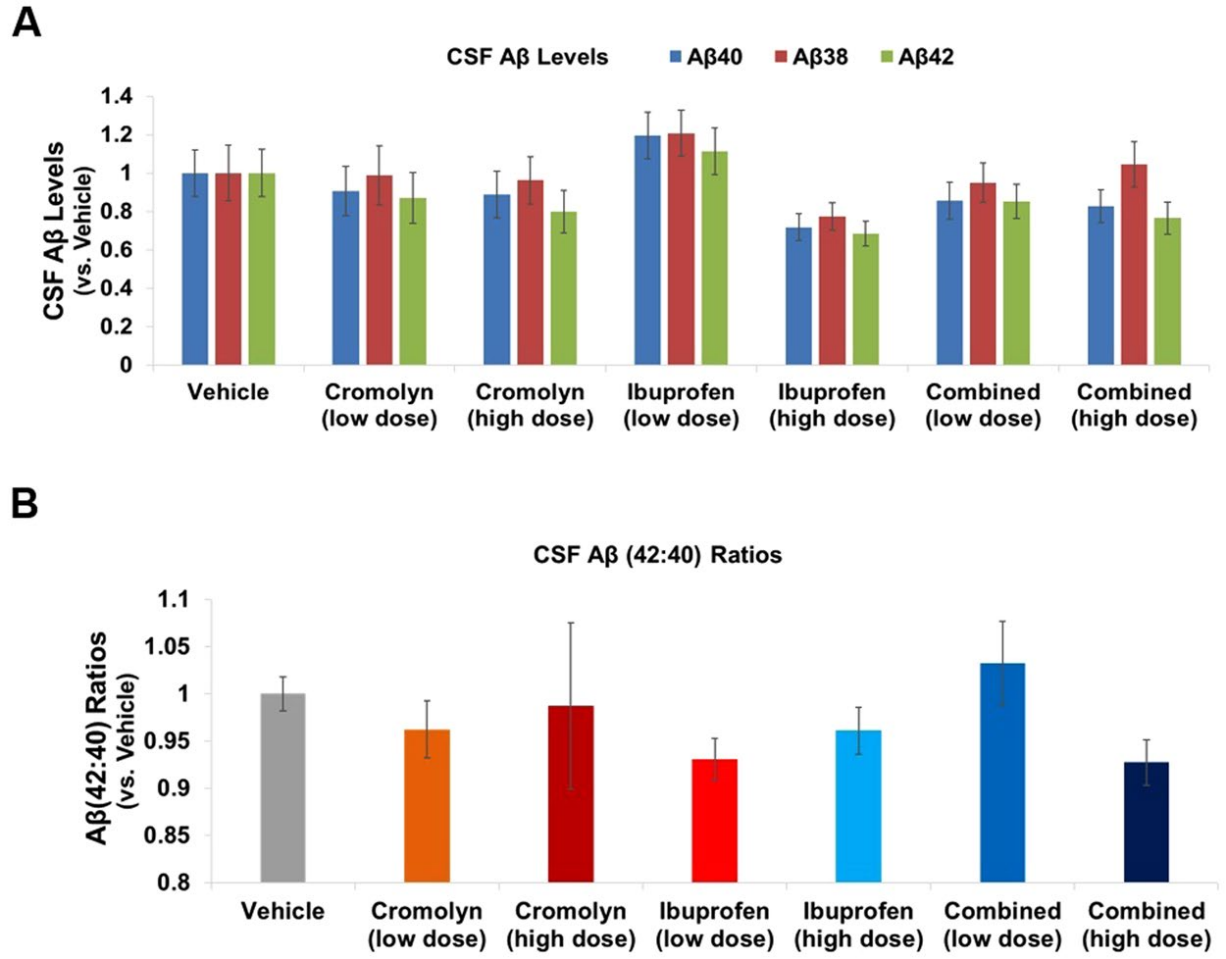
Cromolyn and/or ibuprofen did not significantly influence the levels of Aβ and Aβ42:Aβ40 ratios in mouse CSF samples. (**A**,**B**) MesoScale Aβ-triplex analyses were applied to CSF samples. Differences in Aβ levels (**A**) and Aβ42:Aβ40 ratios (**B**) were analyzed comparing various treatment groups to vehicle. Mean ± SEM; n ≥8 (Table 1); one-way ANOVA with Dunnett’s multiple comparison test.

We next investigated whether cromolyn and ibuprofen, alone, or in combination, affected Aβ levels in plasma (Fig. 3A,B**;** Supplementary Figure 3A,B) and CSF (Fig. 4A,B**;** Supplementary Figure 3C,D), as compared to vehicle. Only low-dose cromolyn, alone, increased the plasma Aβ42:Aβ40 ratios (p < 0.05; Fig. 3B**;** Supplementary Figure 3B). However, this effect was not dose-dependent since high-dose cromolyn, alone, did not exhibit similar effects.

Overall, we showed that cromolyn and ibuprofen, alone, and/or in combination with each other, decreased levels of cerebral TBS-insoluble Aβ40 and Aβ42, and increased soluble brain or plasma Aβ species and Aβ42:Aβ40 ratios (cromolyn, alone). In summary, these findings are suggestive of an anti-aggregation/pro-clearance mechanism of cromolyn and ibuprofen, alone, or in combination with each other on cerebral Aβ depositon.

### Effects of Cromolyn and Ibuprofen on Microglia and Aβ

Acute treatment with cromolyn for one week was previously shown to lead to an increased number of microglia around β-amyloid plaques^6^. We therefore performed a stereological analysis to determine if similar effects could be observed after chronic exposure (12 weeks beginning at 5 months old) with cromolyn and ibuprofen, alone, or in combination with each other. Brains in the vehicle group displayed minimal β-amyloid burden, consistent with previous reports in the Tg2576 mouse model at this age, and no significant changes in the β-amyloid plaque density (Supplementary Figure 4A) or β-amyloid load (Supplementary Figure 4B) were observed across the treatment groups. These findings are likely explained by the fact that 8 month old Tg2576 mice exhibit minimal plaque pathology.

**Figure 5:**
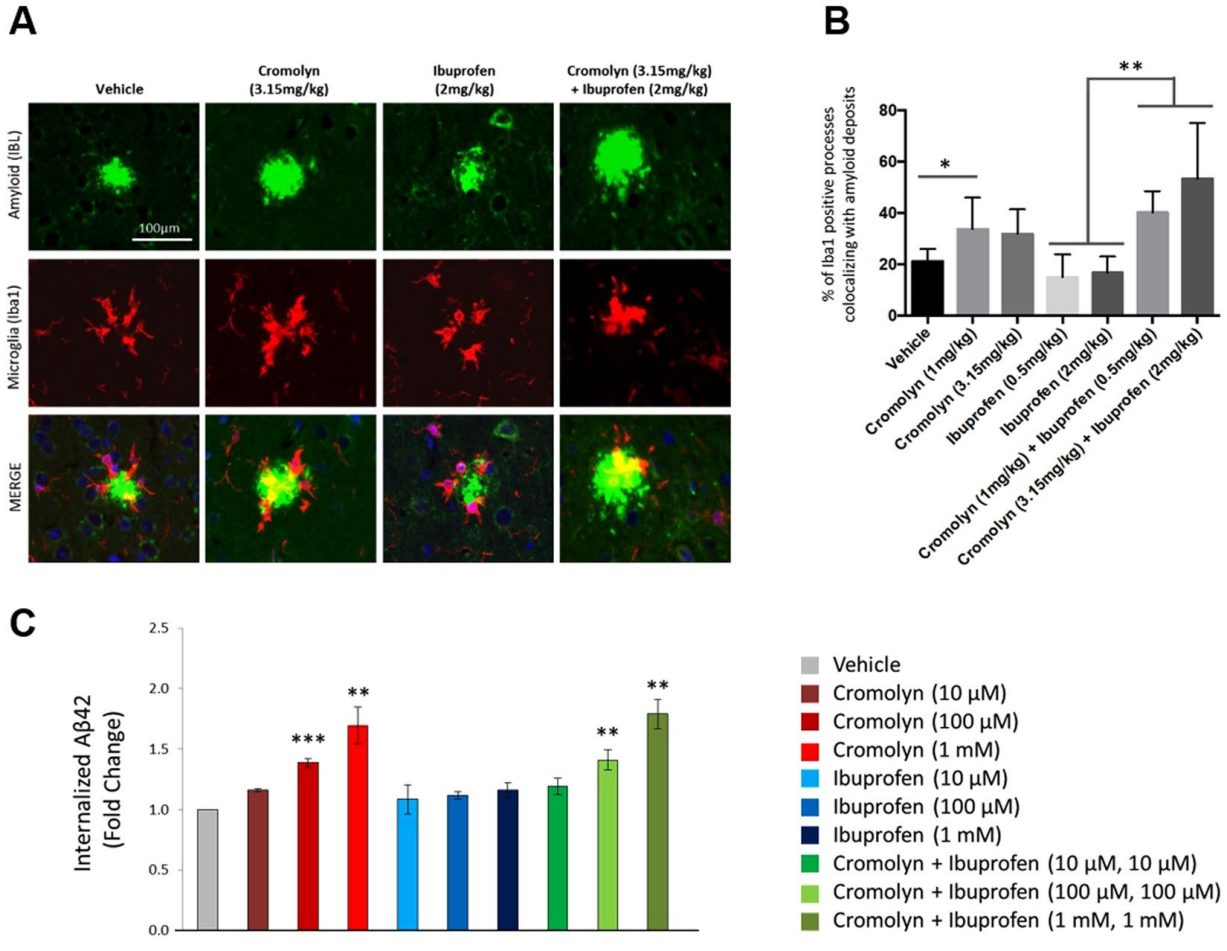
Cromolyn, but not ibuprofen, increased microglial activity in AD mice. (**A**,**B**) Cromolyn increased microglial activity related to β-amyloid deposits in mice. Iba1-positive microglial processes (red) colocalizing with β-amyloid deposits (green) were showed (**A**) and quantified (**B**). We represented pictures of β-amyloid deposits and Iba-1 positive microglia for the highest concentration only from each drug treatment group. Mean ± SEM; *p < 0.05, **p < 0.01, one-way ANOVA with post-hoc Tukey’s multiple comparison test. (**C**) Cromolyn, but not ibuprofen, promoted Aβ42 uptake in BV2 microglial cell culture studies. BV2 microglial cell cultures were treated with cromolyn and/or ibuprofen (10 μM, 100 μM, 1 mM) for 16 hours. Afterwards, cells were incubated with soluble Aβ42 and the compounds for additional 3 hours. After incubation, cells were collected for Aβ ELISA analysis. BV2 microglial cells treated with cromolyn (100 μM, 1 mM), and with combined cromolyn and ibuprofen (100 μM, 1 mM for each compound) exhibited increased Aβ42 uptake levels relative to BV2 microglia treated with vehicle. Mean ± SEM; n = 3; **p < 0.01, ***p < 0.001, one-way ANOVA post-hoc Tukey’s multiple comparison test.

We next assessed the percentage of β-amyloid deposits that overlapped with microglial processes. As shown in the representive images of colocalization of β-amyloid deposits (detected by the Bam10 antibody, *green*) and microglia (detected by the Iba1-specific antibody, *red*) (Fig. 5A), the percentage of β-amyloid deposits occupied by Iba1-positive processes was calculated for each deposit in all animals. Low-dose cromolyn (p < 0.05; vs. vehicle; Fig. 5B) increased the overlap of microglial processes with β-amyloid deposits, while high dose cromolyn exhibited only a trend in this direction. No changes in overlap were observed after treatment with the low or high doses of ibuprofen (Fig. 5B). Combined low-dose and high-dose cromolyn with ibuprofen also significantly increased the overlap of β-amyloid deposits with microglial processes as compared to ibuprofen alone (p < 0.01; Fig. 5B). Overall, our results in the 12-week treatment period were consistent with the previous acute 7-day study suggesting that microglial recruitment and subsequent clearance of Aβ is increased by cromolyn. Treatment with ibuprofen, alone or in association with cromolyn, had no impact on this parameter.

To further explore the effects of cromolyn and ibuprofen, alone, or in combination, we next employed a microglial cell-based assay to assess effects on Aβ42 uptake. Specifically, BV2 murine microglial cell cultures were treated with cromolyn and/or ibuprofen (10 μM, 100 μM and 1 mM, respectively) for 16 hours. Subsequently, cells were incubated with soluble Aβ42 and the compounds for 3 hours. After incubation, cells were collected for ELISA analysis to measure Aβ42 levels. BV2 microglial cells treated with cromolyn, alone (100 μM, 1 mM), and with cromolyn plus ibuprofen (100 μM, 1 mM for each compound), exhibited increased Aβ42 uptake relative to cells treated with vehicle (n = 3; p < 0.001 and p < 0.01, respectively, one-way ANOVA with Tukey’s test; Fig. 5C). In contrast, BV2 microglial cells treated with ibuprofen (10 μM, 100 μM, 1 mM), alone, displayed no change in Aβ42 uptake levels relative to cells treated with vehicle. Collectively, the microglial cell-based studies were consistent with the results of the Tg2576 mouse-based studies. Moreover, the Tg2576 mouse and cell-based studies support the notion that cromolyn decreased TBS-insoluble Aβ levels at least, partially, by promoting a microglial pro-phagocytic state that leads to enhanced clearance of Aβ.

## Discussion

Excessive accumulation and aggregation of Aβ in senile plaques are key pathological events leading to AD-related dementia^2–4^. Numerous pharmacological approaches to prevent and treat AD have targeted Aβ production, accumulation, and/or aggregation, most recently focusing on pre-symptomatic, prodromal, and mildly affected patients^17^. In addition, the neuroinflammatory response driven by both β-amyloid and neuronal cell death has emerged as an important therapeutic target for modifying AD progression, especially for symptomatic AD patients. Here, we studied the effects of cromolyn and the NSAID, ibuprofen, alone, and in combination, on AD pathogenesis in Tg2576 AD mice and microglial cell clearance assays.

Previously, we showed that cromolyn is a strong anti-Aβ aggregation agent and that 1-week treatment decreased Aβ content in AD mice^6^. Here we tested longer term (12 week) treatment in relatively young Tg2576 AD mice with minimal amyloid deposition. We treated these animals with cromolyn and ibuprofen, alone or in combination with each other, via IP injection starting at five months old, for a total of three months. We showed that cromolyn and ibuprofen, alone or in combination with each other, almost abolished longer Aβ peptides (i.e. Aβ40 and Aβ42), while increasing the shorter Aβ38 peptide in brain TBS-insoluble fractions, which serve as the major source of plaque amyloid. (Fig. 1; Supplmental Figure 2). In contrast, we observed increases in various Aβ species in TBS-soluble samples in Tg2576 mice treated with high-dose cromolyn and combined high-dose cromolyn plus ibuprofen (Fig. 2). Equilibrium of soluble and insoluble Aβ plays a critical role in the pathogenesis of AD and thus offers crucial insights in developing an effective AD therapeutic^18,19^. Recently we reported that cromolyn significantly impacted brain Aβ levels in transgenic APPswe/PS1ΔE9-expressing (also known as APP/PS1) mice with abundant cerebral β-amyloid plaques^6^. Our current findings suggest that cromolyn, alone, and combined with ibuprofen decreases insoluble Aβ levels and could potentially attenuate β-amyloid deposition, while increasing soluble pools of Aβ. Moreover, our results support a molecular mechanism for cromolyn that not only decreases cerebral Aβ aggregation but also leads to an up-regulation of plasma Aβ42:Aβ40 ratios perhaps owing to increased export of soluble Aβ42 out of the brain (Fig. 3).

While our previous study implicated anti-aggregation effects of cromolyn on Aβ^6^, they also suggested increased recruitment of microglia to amyloid plaques in Tg2576 AD mice. Our current study showed that cromolyn, but not ibuprofen, significantly promoted microglial cell reactivity to Aβ deposition. Moreover, in microglial cell-based assays, we showed that cromolyn was able to upregulate microglial-uptake of Aβ in a dose-dependent manner (Fig. 5). Together, these findings suggest that cromolyn may not only prevent aggregation of Aβ but also induce the transition of microglia cells from a pro-inflammatory/neurotoxic to a pro-phagocytic/neuroprotective activation state. These findings carry implications for the potential use of cromolyn, alone, or in combination with ibuprofen in the treatment of AD. If cromolyn were only affecting aggregation of Aβ it would likely need to be used in subjects positive for amyloid deposition prior to symptoms, prodromally, or in very mild, early stage AD patients. The observed effects of cromolyn (alone, or in combination with ibuprofen) on recruitment of microglia to plaques and enhanced microglial uptake of Aβ, suggest an additional role for cromolyn in the potential ability to convert microglial activation state from one favoring neuroinflammation to one promoting phagocytosis of Aβ state. The molecular mechanisms by which cromolyn regulates microglial activity in Aβ42 uptake will require future studies.

In summary, AD transgenic mouse model and microgial cell-based studies of cromolyn, alone, or in combination with ibuprofen, together with the favorable safety profile of these FDA-approved drugs, strongly support their potential for effectively treating and preventing AD. Given the effects of these drugs on both β-amyloid deposition and microglial activation state, our findings suggest that cromolyn, alone, or in combination with ibuprofen, could serve as an effective AD therapeutic in both early and later stage cases of the disease. Cromolyn and ibuprofen (ALZT-OP1) are currently in a phase three clinical trial (COGNITE) for the treatment of AD (http://www.alzforum.org/therapeutics/alzt-op1; https://clinicaltrials.gov/ct2/show/NCT02547818?term=ALZT+OP1&rank=2).

## Material and Methods

### Animals

Animal housing, handling and procedures were conducted under the protocols and/or guidelines approved by the Institutional Animal Care and Use Committee (IACUC) of Cephrim Biosciences, Inc. Male, transgenic APPswe mice (Tg line 1349; B6:SJL-Tg2576Kha), aged between 11 to 12 weeks, were purchased from Taconic and previously reported^15,16^. Upon delivery, they were maintained in individual housing. Animals were randomly assigned into one of the seven treatment groups and then were given an extra 2 months to grow in the facility in order to reach 5 month-old age for treatments. The treatment solutions (Table 1) were prepared by and transferred from AZ Therapeutics and stored at 4 °C during the study. All treatments were conducted three times a week intraperitoneally (IP) with the injection volume of 3 mL/kg (Table 1). As additional control for AD β-amyloid pathology, we also obtained additional young Tg2576 mice and sacrificed 5 days upon delivery (11–12 weeks old), followed by tissue harvesting, processing analysis.

### Antibodies

The mouse monoclonal Bam10 antibody was utilized to detect β-amyloid plaques through immunohistochemistry (Sigma-Aldrich; 1:500). The rabbit polyclonal anti-Iba-1 antibody was used to detect microglia (Wako Chemicals; 1:500). The secondary antibodies included the goat anti-mouse Alexa Fluor 488 and goat anti-rabbit Alexa Fluor 568-conjugated antibodies (ThermoFisher; 1:1,000). The DAPI-containing mounting media was from Dako (Carpinteria, CA).

### Tissue harvest and protein extraction

We processed the tissue and performed post-mortem analysis according to methods previously reported^6,20–23^. Mice were transcardiacally perfused with ice cold phosphate-buffered saline buffer solution (PBS) and the brain was removed. Hemispheres were then weighed and homogenized with 5 × volume tris-buffered saline (TBS) buffer solution (mL; based on weight in mg) containing 1 × Halt protease inhibitor cocktail (Thermo Fisher Scientific) and 5 mM EDTA. This step was utilized to generate total TBS extracted brain samples (TTBS), which were then processed by ultracentrifugation at 100,000 g for 1 hr at 4 °C. The supernatant was collected as TBS-soluble brain samples (STBS). The pellet was further extracted in 400 μL of 70% formic acid (FA) followed by homogenization and subsequent ultracentrifugation at 100,000 g for 1 hr at 4 °C. The supernatant was collected as TBS-insoluble, or also known as FA-soluble samples. Furthermore, we processed TTBS by adding 3X volume M-PER (Mammalian Protein Extraction Reagent) (Thermo Fisher Scientific) with 1X Halt protease inhibitor cocktail, followed by centrifugation at 10,000 g for 20 minutes at 4 °C. The supernatant was transferred to a new Eppendorf tube and collected as total proteins (or MPER-processed TBS), which contained membrane proteins, e.g. APP-FL and APP-CTFs. BCA analysis was performed on total proteins to determine their concentrations^24,25^.

### MesoScale Aβ analysis

Levels of Aβ peptides were determined using protocols suggested by the manufacturer and have been previously reported^23,26–29^. For this purpose, we utilized an electrochemi-luminescence-based multi-array method on the platform of the Quickplex SQ 120 system (by the Meso Scale Diagnostics LLC). The 6E10 MesoScale Aβ 3-plex kits were utilized to detect the Aβ triplex peptides (i.e. Aβ[40/38/42]) in a 96-well-based assay system. Briefly, 96-well plates were blocked with diluents provided by the manufacturer with shaking for 1 hour at room temperature (RT). Next, the protein samples and the MesoScale protein standards were resuspended in the manufacturer-supplied detection antibodies. The mixed solutions were then placed on a shaker for 2 hours at RT, followed by washing and adding of the reading buffer. Next, the electrochemi-luminescence signals were captured by the MesoScale SQ 120 system and robust signals were obtained for all samples and standard proteins. Finally, sample Aβ40, Aβ38 and Aβ42 levels were analyzed using the MesoScale protein standards. TBS-soluble Aβ levels were further normalized to sample total protein concentrations. For TBS-insoluble samples whose Aβ levels were even lower than the lowest protein standard in some treatment groups, imputation values were calculated by taking half the minimum value of each group for inclusion.

### Immunohistochemistry (IHC)

Serial sagittal paraffin embedded sections were cut at 5μm and two sections were placed on each slide. One of ten set slices were immunostained with a rabbit anti-human Aβ antibody (Immuno- Biological Laboratories, 1:500) for amyloid plaques, followed by biotinylated goat anti-rabbit secondary antibody (Vector Laboratories) and developed using the ABC Elite and DAB kits (Vector Laboratories). Images were obtained using an Axioscope A1 microscope (Carl Zeiss) and were analyzed to determine the amyloid load using SWIFT M1000-D under the 10 × objective (after background correction to avoid uneven lighting). To analyze the colocalization and quantify the overlap between amyloid plaques and microglial cells, sagittal sections were blocked in PBS with 10% NGS, and immunostained overnight with a mouse anti-human amyloid antibody (Bam10, Sigma-Aldrich, 1:500) for amyloid plaques and a rabbit anti-Iba-1 for microglia (Wako Chemicals, 1:500). The next day, the sections were thoroughly washed in PBS, incubated with 1:1000 goat anti-mouse Alexa Fluor 488 and goat anti-rabbit Alexa Fluor 568-conjugated antibodies (ThermoFisher) for 2 h at room temperature, washed again in PBS, and coverslipped with fluorescent mounting media containing DAPI (Dako, Carpinteria, CA). Tile-scan images were collected on a Zeiss Axio Imager Z epifluorescence microscope equipped with AxioVision software and modified for automated acquisition of entire cortices, using either 5 × or 20 × objectives. Exposure times for each specific immunostaining were maintained unchanged between each slice imaged. Quantification of the amyloid load and amyloid density in the cortex was done with ImageJ (NIH) using the “Analyze Particles” function after background subtraction and application of a median filter. The percentage of amyloid deposits covered by Iba1 positive processes was calculated by applying a threshold to each amyloid deposit image, selecting the corresponding region of interest (“plaque”) and applying this “plaque mask” to the Iba-1 channel in order to identify the area of amyloid colocalizing with Iba-1 signal.

### Aβ uptake assay

BV2 microglial cells were cultured in DMEM containing 10% heat-inactivated fetal bovine serum, 2mM L-Glutamine and 1% penicillin/streptomycin (Life Technologies). BV2 cells were treated for 16 hours with cromolyn (10 μM, 100 μM, 1 mM) and/or ibuprofen (10 μM, 100 μM, 1 mM). Subsequently, cells were incubated with 2 μg/ml Aβ42 (AnaSpec) and the above-mentioned compounds in serum-free DMEM medium for 3 hours. Afterwards, cells were extensively washed with PBS and were lysed in RIPA lysis buffer (EMD Millipore) supplemented with EDTA-free protease inhibitors (Roche), Halt phosphatase inhibitor cocktail (ThermoFisher Scientific) and 2 mM 1,10 phenantroline (Sigma). Lysates were centrifuged at 12,000 *g* at 4 °C for 15 minutes and supernatants were collected. Aβ42 levels were measured in the supernatants using Aβ42 ELISA kit (Wako Chemicals) and normalized to total protein concentration that was assessed by BCA method (Pierce).

### Data analysis

Protein levels from various treatments were compared to the vehicle treatment. Protein levels were demonstrated as means ± SEM in each treatment group. P values < 0.05 were considered statistically significant. One-way ANOVA with Dunnett’s multiple comparison test was used to compared the differences between drug-treatment group to vehicle for MSD Aβ studies. One-way ANOVA with Tukey’s test was used to compare the difference among multiple treatments for IHC and microglial cell-based studies.

## Electronic supplementary material

Supplementary Information
