# Supplementary material for: Cromolyn Reduces Levels of the Alzheimer’s Disease-Associated Amyloid β-Protein by Promoting Microglial Phagocytosis
Source: Sci Rep. 2018 Jan 18;8:1144. doi: 10.1038/s41598-018-19641-2 (PMC5773545; doi:10.1038/s41598-018-19641-2)
Supplement: Supplementary file 1 — Supplementary Information [file 41598_2018_19641_MOESM1_ESM.pdf]

Supplementary Information – for the manuscript titled below and author list.

Cromolyn Reduces Levels of the Alzheimer's Disease-Associated Amyloid β-Protein by Promoting Microglial Phagocytosis

Can Zhang[1], Ana Griciuc[1], Eloise Hudry[2], Yu Wan[1], Luisa Quinti[1], Joseph Ward[1], Angela E. Forte[1], Xunuo Shen[1], ChongZhao Ran[3], David R. Elmaleh[3], Rudolph E. Tanzi[1]

[1]Genetics and Aging Research Unit,
MassGeneral Institute for Neurodegenerative Diseases (MIND),
Department of Neurology,
Massachusetts General Hospital and Harvard Medical School,
Charlestown, MA, 02129-2060, USA

[2]Alzheimer's Disease Research Unit,
MassGeneral Institute for Neurodegenerative Diseases (MIND),
Department of Neurology,
Massachusetts General Hospital and Harvard Medical School,
Charlestown, MA, 02129-2060, USA

[3]Department of Radiology,
Massachusetts General Hospital and Harvard Medical School,
Charlestown, MA, 02129-2060, USA

Supplementary Figures:

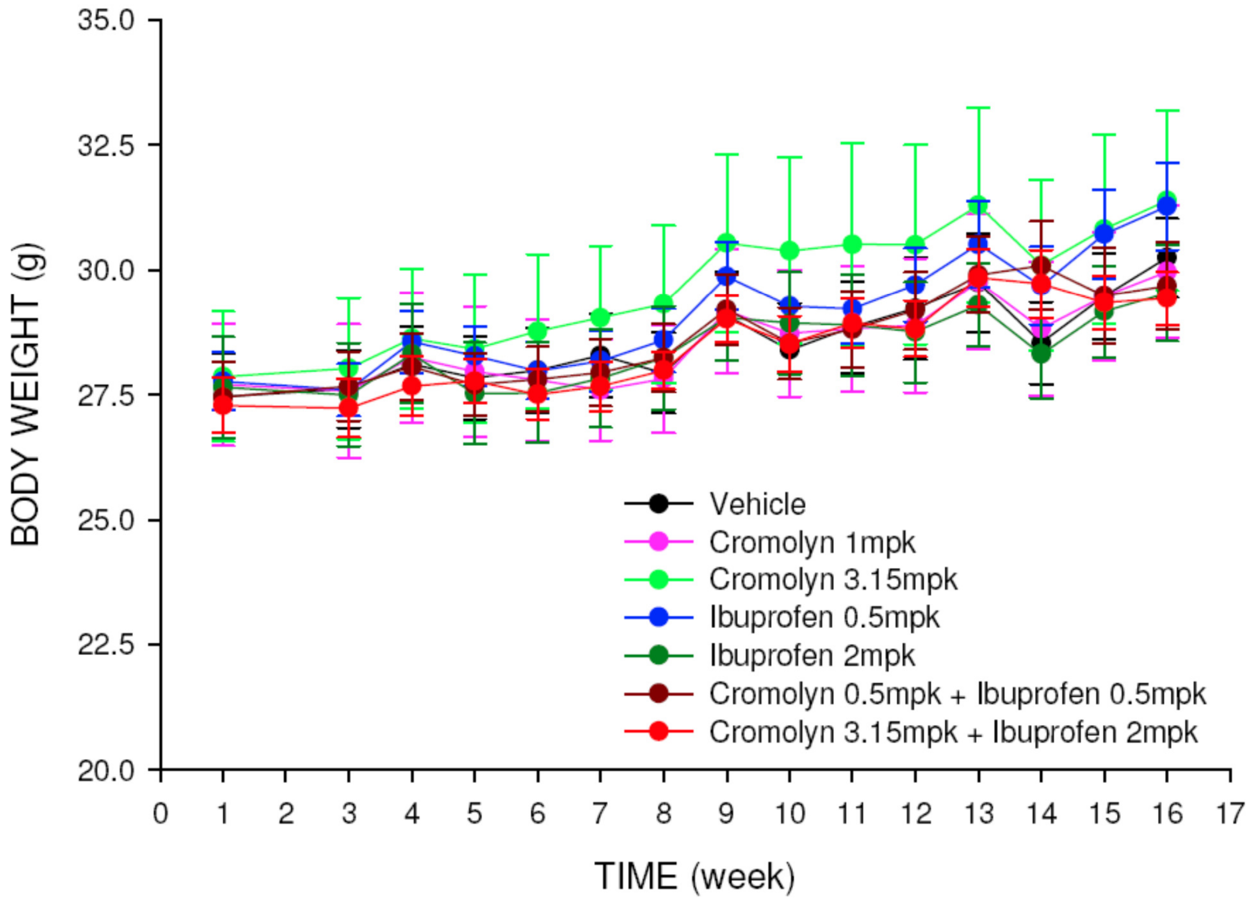

Supplementary Figure 1: Body weight of experimental treatment groups. No differences in mouse body weight were observed throughout the treatment period.

Supplementary Figure 2:

A

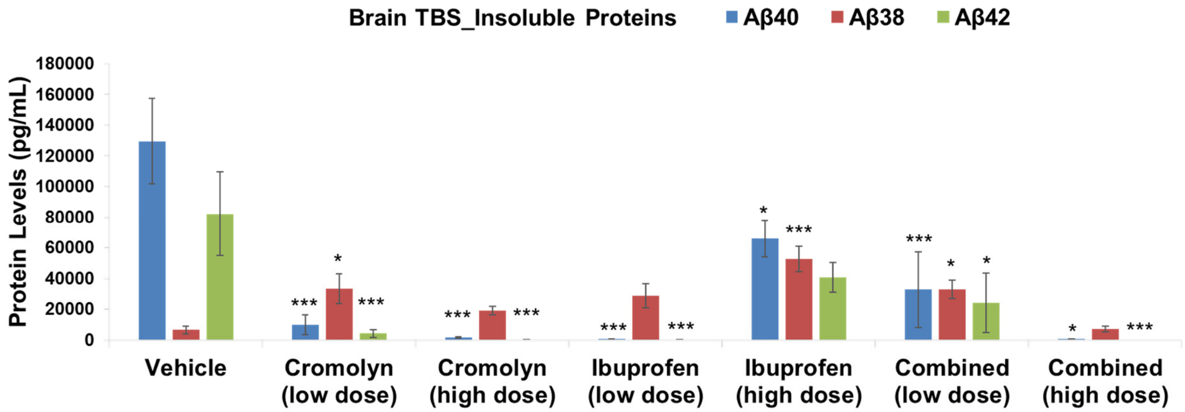

B

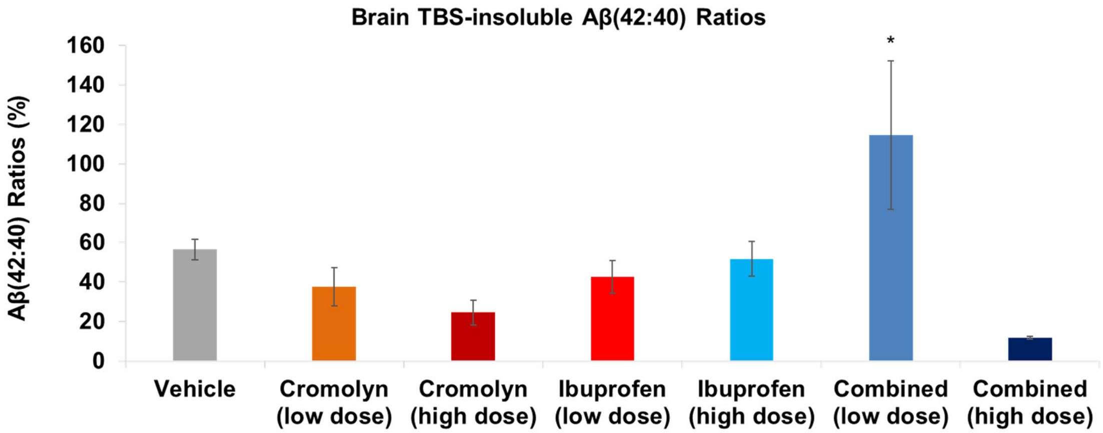

C

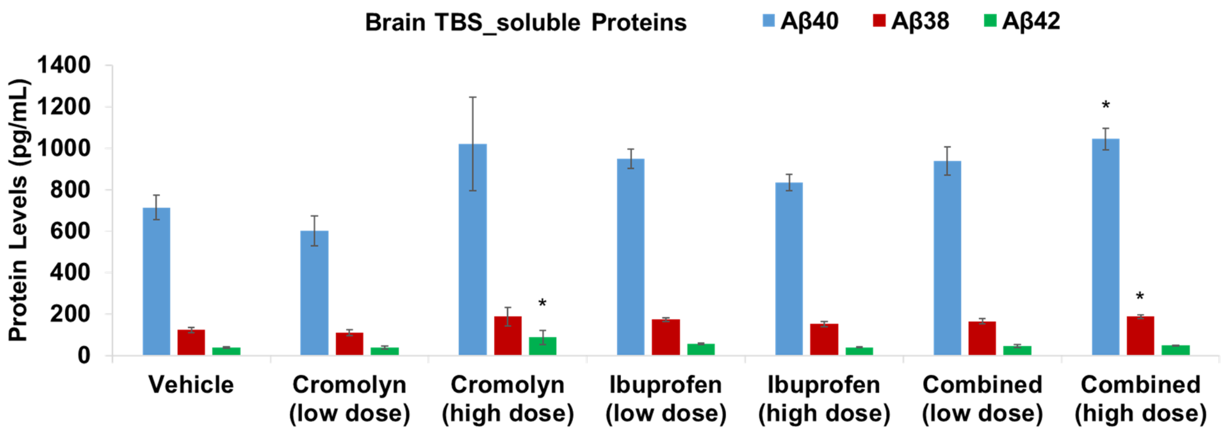

D

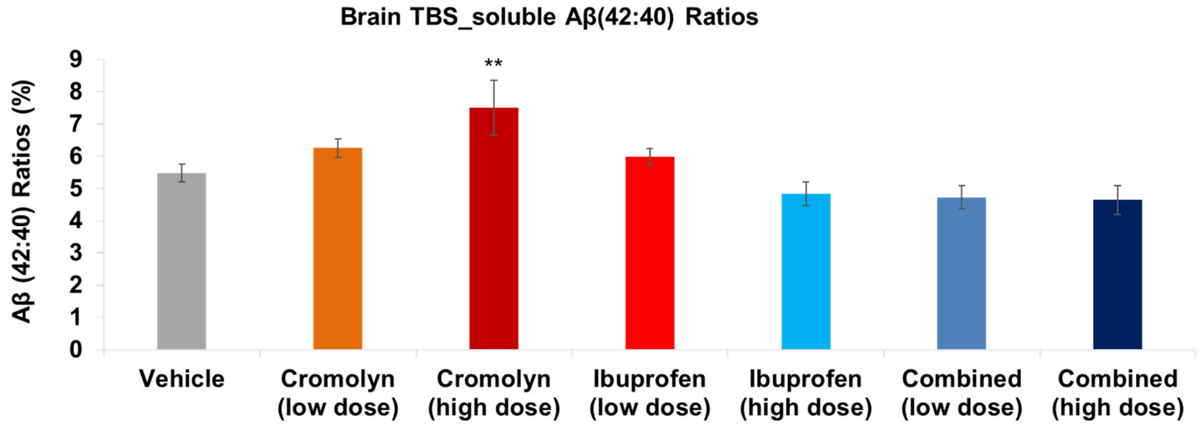

<u>Supplementary Figure 2:</u> **Analysis of Aβ levels and Aβ42:Aβ40 ratios in brain TBS-soluble and TBS-insoluble samples**. Differences in Aβ levels and Aβ42:Aβ40 ratios were assessed comparing treatment groups to vehicle.

A-B. Aβ levels (**A**) and Aβ42:Aβ40 ratios (**B**) in brain TBS-insoluble samples.

C-D. Aβ levels (**C**) and Aβ42:Aβ40 ratios (**D**) in brain TBS-soluble samples.

A

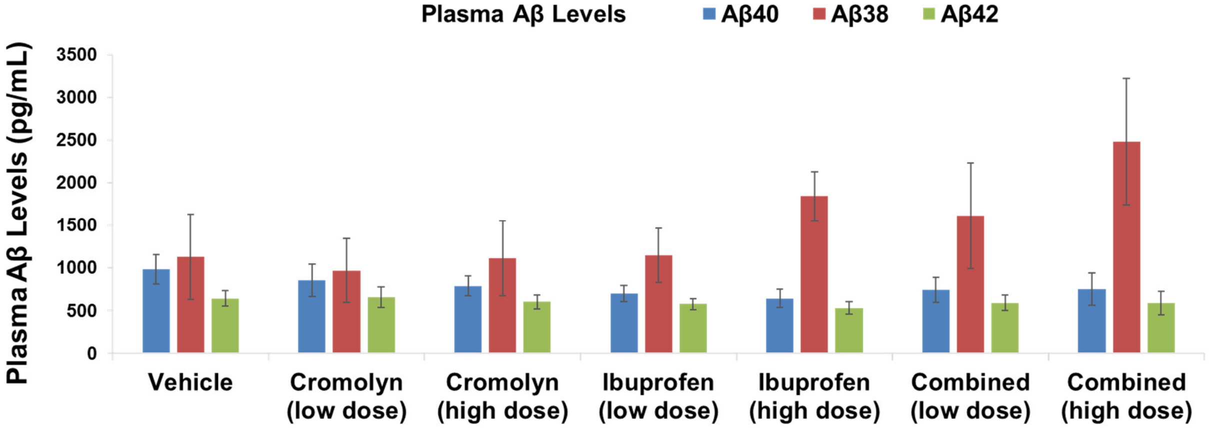
Plasma Aβ Levels
Aβ40
Aβ38
Aβ42
Plasma Aβ Levels (pg/mL)
3500
3000
2500
2000
1500
1000
500
0
Vehicle
Cromolyn (low dose)
Cromolyn (high dose)
Ibuprofen (low dose)
Ibuprofen (high dose)
Combined (low dose)
Combined (high dose)

B

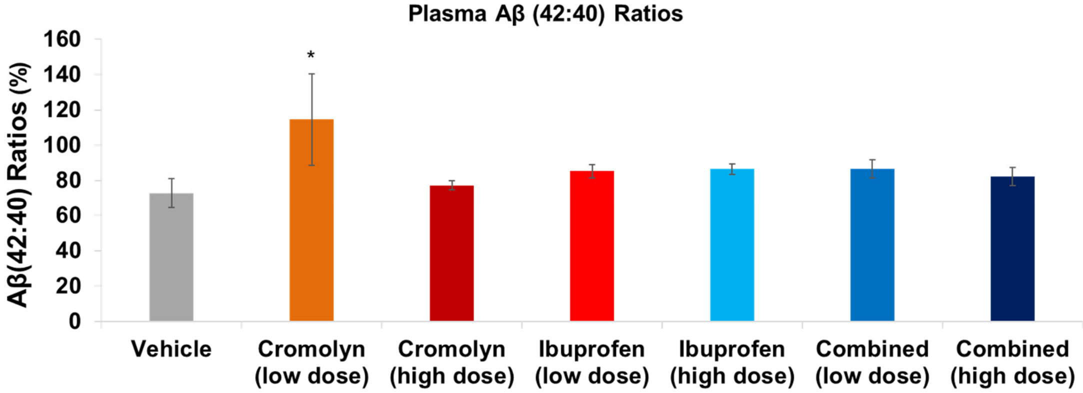
Plasma Aβ (42:40) Ratios
Aβ(42:40) Ratios (%)
160
140
120
100
80
60
40
20
0
*
Vehicle
Cromolyn (low dose)
Cromolyn (high dose)
Ibuprofen (low dose)
Ibuprofen (high dose)
Combined (low dose)
Combined (high dose)

C

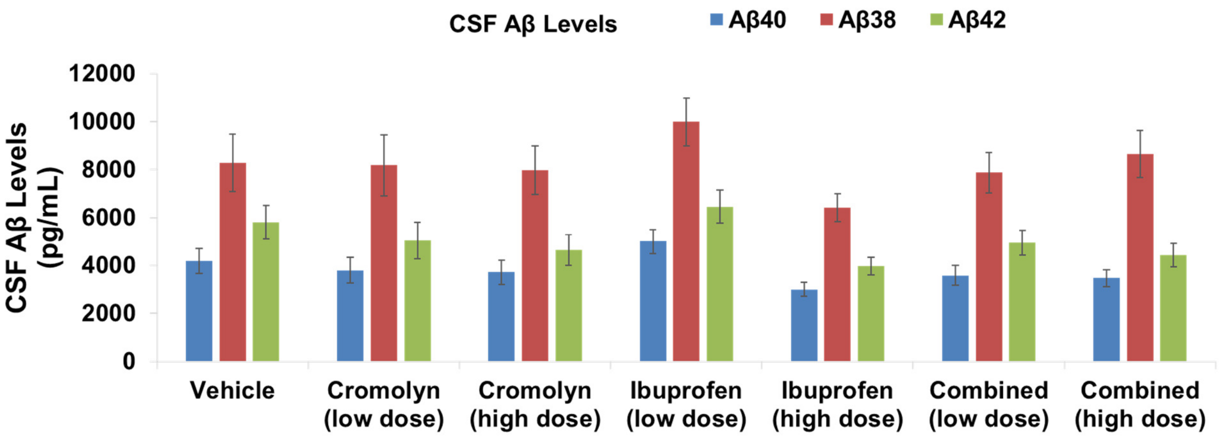

D

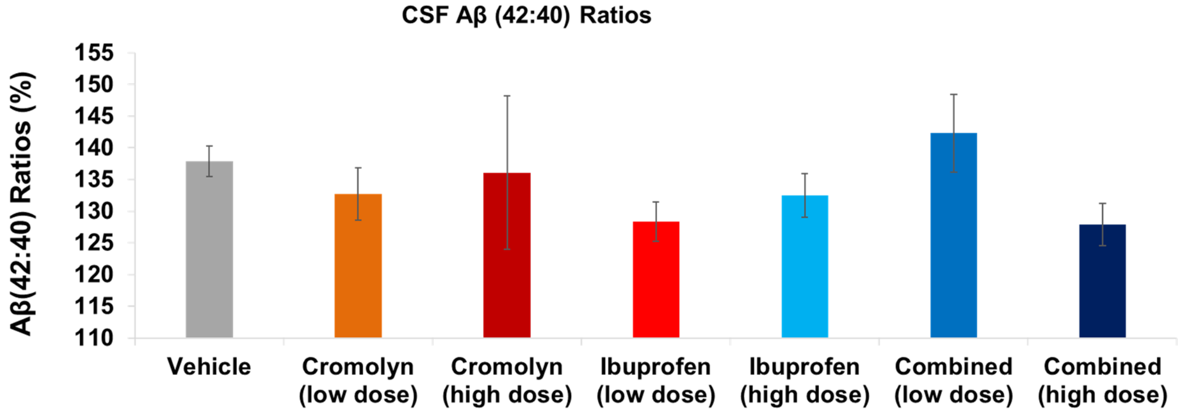

<u>Supplementary Figure 3:</u> Analysis of Aβ levels and Aβ42:Aβ40 ratios in plasma and CSF samples. Differences in Aβ levels and Aβ42:Aβ40 ratios were assessed comparing treatment groups to vehicle.

A-B. Aβ levels (**A**) and Aβ42:Aβ40 ratios (**B**) in plasma samples.

C-D. Aβ levels (**C**) and Aβ42:Aβ40 ratios (**D**) in CSF samples.

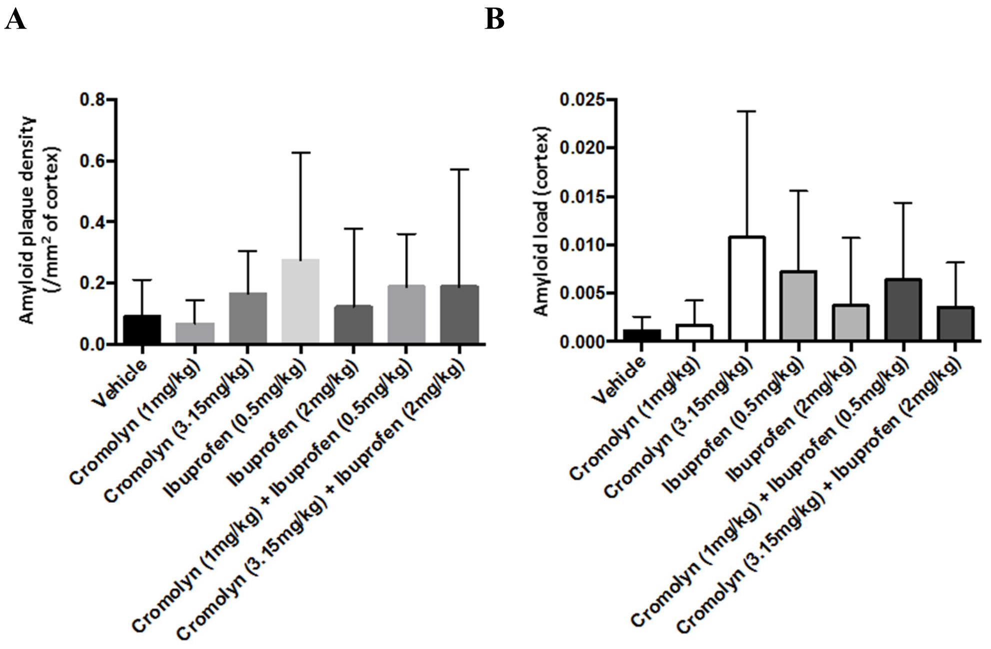

<u>Supplementary Figure 4.</u> **Three month treatments with cromolyn, ibuprofen or a combination of both did not impact β-amyloid plaque levels in 8 month-old Tg2576 mice.**

A-B. Cromolyn sodium (1.05 or 3.15 mg/kg) or ibuprofen (0.5 or 2 mg/kg) were administered via intraperitoneal injection for 3 months, either alone or in combination. Treatment started at 5 months old, when cerebral β-amyloid deposition has not yet started, and ended when the mice were 8 months old with minimal β-amyloid burden. Stereological analysis revealed no significant change in the plaque density (***A***) or the β-amyloid load (***B***) across the experimental groups (n = 8-10 mice/group; one-way ANOVA and post-hoc Tukey's multiple comparison test).
